# Disulfide rebridging platforms for site-specific ADC conjugation

**DOI:** 10.1039/d6cb00121a

**Published:** 2026-08-11

**Authors:** László Petri, Írisz K. Kovács, Blanka J. Kiss, György M. Keserű

**Affiliations:** a Medicinal Chemistry Research Group, HUN-REN Research Centre for Natural Sciences, 2 Magyar tudósok krt Budapest 1117 Hungary keseru.gyorgy@ttk.hu; b Institute of Chemistry, ELTE Eötvös Loránd University, 1-3. Egyetem tér 1053 Budapest Hungary; c Department of Organic Chemistry and Technology, Budapest University of Technology and Economics, 4 Szt. Gellért tér Budapest 1111 Hungary; d Department of Applied Biotechnology and Food Science, Budapest University of Technology and Economics, 4 Szt. Gellért tér Budapest 1111 Hungary

## Abstract

Antibody–drug conjugates (ADCs) have emerged as a transformative class of targeted therapeutics, combining the exquisite specificity of monoclonal antibodies with the potency of small-molecule cytotoxic payloads. While numerous strategies exist to generate ADCs, site-specific conjugation methods have gained prominence due to their ability to produce homogeneous constructs with defined drug–antibody ratios, improved stability, and predictable pharmacokinetics. Among these, disulfide rebridging chemistries have recently attracted considerable attention as an elegant and versatile platform for precise antibody modification. By exploiting native interchain disulfide bonds, rebridging approaches restore structural integrity while introducing functional handles for payload attachment, enabling the generation of reproducible and structurally well-defined ADCs from native antibodies. This review provides a systematic and comprehensive overview of the chemical methodologies developed for disulfide rebridging, tracing the evolution of rebridging reagents and strategies from early maleimide and bissulfone scaffolds to modern dibromopyridazinediones, and other bridging frameworks. Beyond chemistry, we discuss the biological implications of rebridged ADCs, including their translational potential, supported by emerging preclinical and clinical data. Providing a dual chemical and biological perspective, this review aims to serve as a practical guide for researchers seeking to navigate the expanding landscape of site-specific ADC development, ultimately contributing to the rational design of next-generation targeted therapeutics.

## Abstract 2

Disulfide rebridging chemistries have recently attracted considerable attention as a versatile platform for site-specific antibody modification and generating homogeneous antibody–drug conjugates (ADCs).

## Graphical abstract

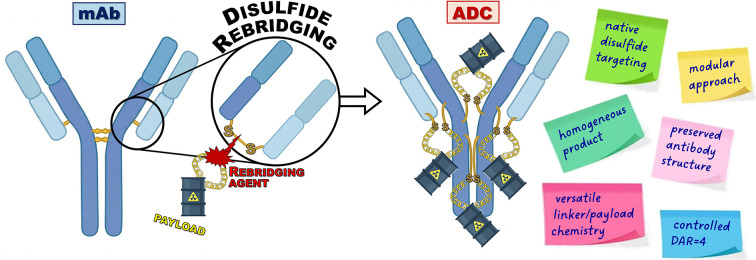

## 1. Introduction

**Fig. 1:**
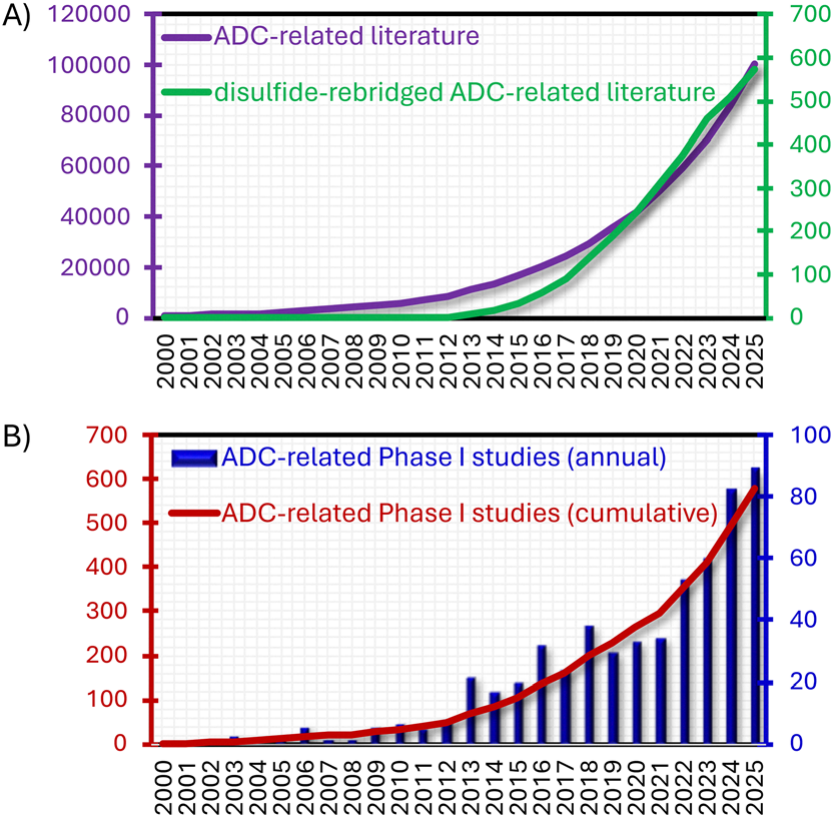
The growth of ADC research and its translation into early clinical development. A) Number of literature records retrieved from the Google Scholar database for the period 2000–2025 (accessed 16 March 2026). The purple curve represents the cumulative count of ADC-related publications, while the green curve shows publications specifically related to disulfide rebridged ADCs. (B) Trends in the clinical translation of ADCs based on data retrieved from the ClinicalTrials.gov database (2000–2025, accessed 16 March 2026). Blue columns indicate the annual number of newly initiated Phase I clinical studies (including early Phase I, Phase I, and combined Phase I/II trials) involving ADCs, while the red curve represents the cumulative number of such trials initiated in this period.

Antibody–drug conjugates (ADCs) have emerged as one of the most transformative therapeutic modalities in modern precision oncology, fundamentally redefining the traditional paradigm of targeted cancer treatment.^1–4^ This strategy, often described metaphorically as a “biological missile,” combines the targeting precision of biologics with the cell-killing strength of cytotoxic chemotherapy.^5,6^ Over the past two decades, the field has entered an unprecedented phase of scientific and clinical acceleration, with 21 ADCs receiving regulatory approval worldwide so far, and an expansive clinical pipeline continuing to grow rapidly.^7–9^ Consequently, ADC development is currently experiencing an explosive phase of scientific innovation and clinical translation (Fig. 1). However, despite these remarkable milestones, key challenges remain, with some of them related to product heterogeneity mostly associated with conventional bioconjugation approaches.^10–15^ Consequently, addressing these fundamental limitations necessitates the adoption of advanced bioconjugation strategies, thereby shaping the development of next-generation ADCs.

At the molecular level, ADCs are modular constructs composed of three canonical elements: a monoclonal antibody that functions as a targeting vector, a chemical linker that connects the antibody to the drug, and a highly potent cytotoxic payload that ultimately mediates tumor cell killing. While optimization of these three components remains fundamental, the bioconjugation strategy employed to assemble them is increasingly recognized as a critical determinant of therapeutic performance.^16^ Precise control over the drug-to-antibody ratio (DAR) and its distribution remains a central challenge in ADC development. Conventional lysine- and cysteine-targeting strategies typically yield highly heterogeneous mixtures with variable DAR and positional isomers, alongside significant instability in the case of maleimide-based linkages. Such heterogeneity can affect pharmacokinetics, stability, and toxicity: excessive DAR values increase hydrophobicity and accelerate clearance, whereas insufficient drug loading compromises efficacy.^14^ Consequently, the ability to generate structurally defined conjugates with controlled DAR has emerged as a central objective in ADC design, driving the rise of site-specific conjugation methodologies that afford improved molecular homogeneity, pharmacokinetic stability, and more predictable clinical behavior.^7,17^ In particular, maleimide conjugates formed *via* reduction of interchain disulfides are prone to deconjugation and thiol exchange in circulation, resulting in payload loss (*e.g.*, DAR decrease from 4 to <3.5 within 120 h) and the formation of lower DAR species and free antibody.^18^ Recently,^19^ disulfide rebridging approaches based on cysteine bis-alkylation of reduced antibodies have been shown to overcome these limitations by restoring covalent connectivity while enabling site-selective and homogeneous modification of native antibodies without protein engineering efforts. This strategy affords predominantly DAR = 4 conjugates (≥78%) with narrow distributions and markedly improved stability (DAR ∼3.9 after 120 h in serum conditions), while fully preserving antigen binding.

Importantly, in this specific ADC system, based on a PEGylated valine–citrulline-linked MMAE payload, systematic comparison of isolated DAR species demonstrated that lower DAR variants (1–2) exhibited limited *in vivo* efficacy, whereas higher and well-defined DAR 3–4 conjugates provided superior tumor control, with the DAR 4 ADC achieving complete tumor regression in xenograft models. Collectively, these findings highlight that controlling both DAR and conjugation homogeneity is essential and establish disulfide rebridging as a robust chemical solution that addresses key limitations of classical conjugation strategies, thereby motivating the development of next-generation site-selective ADC conjugation methodologies. Within this evolving landscape, disulfide rebridging strategies have garnered significant attention as an elegant and versatile approach for site-specific antibody modification. Although disulfide rebridging chemistries remain relatively young compared with classical conjugation techniques, over the past decade, a diverse repertoire of reagents has emerged to address challenges associated with stability, selectivity, and scalability. These innovations exemplify the broader shift in ADC design toward precision bioconjugation strategies that nominates disulfide rebridging not merely as an alternative, but as a platform capable of enabling next-generation, structurally defined ADCs. Disulfide rebridging chemistries are particularly compatible with modular ADC synthesis workflows. Convergent “stick-and-click” strategies^18^ decouple bioconjugation from linker-payload attachment, allowing rapid combinatorial exploration of antibody scaffolds, linkers, and payload classes. Rebridging reagents consistently deliver high conjugation efficiency, reproducible DAR profiles, and preserved antigen-binding affinity, while facilitating high-throughput library generation and parallel optimization. Additionally, late stage coupling of the payload reduces handling of highly cytotoxic compounds that simplifies purification workflows and improves safety in both laboratory and industrial settings. In this review, we systematically explore the chemical methodologies for preparing ADCs *via* disulfide rebridging, providing a comprehensive perspective on the evolution of chemical strategies followed by therapeutic implementations.

## 2. General aspects of disulfide rebridging

In principle, disulfide rebridging relies on bifunctional electrophiles that react with the two cysteine thiols available after reduction of native interchain disulfide bonds, replacing the natural disulfide with a synthetic covalent bridge equipped with a handle for functional payloads. Although the original disulfide bond is not reconstructed, efficient rebridging can preserve native-like IgG connectivity and overall antibody architecture. Mechanistically, one cysteine first reacts with the reagent to form a mono-adduct, followed by rapid intramolecular capture of the second proximal thiol to reconstruct interchain linkage. The efficiency of this second step is critical: slow bridge closure increases the likelihood of hydrolysis, intermolecular reactions, or cysteine scrambling, leading to half-antibody species, misbridged products, or heterogeneous higher-order assemblies. Consequently, successful rebridging depends not only on thiol selectivity, but also on reaction kinetics, linker geometry, rigidity, and compatibility with the native disulfide topology. Because the synthetic linker becomes an integral structural element of the conjugate, its physicochemical properties can additionally influence stability, aggregation, hydrophobicity, and pharmacokinetic behavior of the final ADC.^15^

Densitometric analysis of SDS-PAGE gel bands is frequently used to estimate the relative abundance of intact and half-antibody species, as well as to provide approximate assessments of rebridging efficiency or conjugation conversion. In this review, conversion values are based either on densitometric analyses reported in the cited literature or on densitometric values determined by us from published SDS-PAGE gel images. Notably, such estimates should be interpreted with caution, as they provide approximate rather than definitive quantitative measurements due to inherent limitations of the method. Additionally, half-antibody formation should not automatically be interpreted as a failure of rebridging. To date, there is no evidence indicating that these species have a detrimental impact on the antibody's overall profile.^20^ Moreover, conventional cysteine–maleimide conjugation strategies, widely used in clinically approved ADCs, also generate mixtures containing HL, HHL, HH, and free heavy (H) and light (L) chain species, yet these products generally retain excellent biological performance due to strong noncovalent interchain interactions within IgGs. Similarly, rebridged HL (half-antibody) species often remain associated as intact IgG-like assemblies under native conditions, despite appearing dissociated in denaturing SDS-PAGE or MS analyses. Indeed, ELISA and SEC analyses frequently demonstrate preserved antigen binding and solution stability even in the presence of detectable half-antibody populations. Therefore, the detection of half-antibody species should be interpreted carefully, as their analytical appearance does not necessarily reflect a loss of functional antibody integrity. Nevertheless, the presence and abundance of half-antibody species should be monitored, since a high proportion may also indicate incomplete or inefficient rebridging and reduced restoration of the native disulfide architecture. Therefore, half-antibody formation should always be evaluated in the context of the specific rebridging strategy, analytical method, and biological performance of the resulting ADC, rather than being considered an isolated measure of conjugation success. Consequently, successful rebridging should be evaluated primarily by preservation of functional IgG architecture and biological activity rather than by the complete reconstruction of full-antibody species alone.

Beyond structural integrity, the key quantitative descriptor of conjugation quality is the drug-to-antibody ratio (DAR), or fluorophore-to-antibody ratio (FAR) for diagnostic conjugates, which defines the average number of payload molecules attached per antibody. DAR is commonly determined by hydrophobic interaction chromatography (HIC),^21^ UV-vis spectroscopy,^22^ or mass spectrometry (MS),^23^ the latter providing the most reliable assessment because it directly resolves individual conjugated species and detects incomplete conjugation, scrambling, or over-functionalization. Clinically successful ADCs typically exhibit average DAR values around 4, representing the optimal balance between potency and the increased hydrophobicity, aggregation, instability, and accelerated clearance associated with higher payload loading. Accordingly, disulfide rebridging offers a major advantage because selective targeting of the four interchain disulfides of IgG1 antibodies^24^ enables controlled generation of relatively homogeneous DAR 4 conjugates with substantially reduced heterogeneity compared with stochastic lysine or conventional cysteine conjugation methods. Nevertheless, DAR values exceeding 4 are frequently observed and may arise from several sources, including analytical overestimation, overreduction exposing additional cysteines, incomplete bridge closure, or structural heterogeneity of the antibody itself.^25^ Nevertheless, degree-of-labeling (DOL) values exceeding 4 should always be interpreted with caution, as they generally indicate over-conjugation rather than controlled one-to-one rebridging of the interchain disulfides. Although higher DAR species may initially appear advantageous because of increased payload delivery, they are generally more hydrophobic, aggregation-prone, and less stable, often resulting in poor pharmacokinetic and therapeutic performance. Consequently, recent ADC development prioritizes controlled and homogeneous DAR distributions rather than maximal payload loading alone. Evaluation of rebridging methodologies therefore requires consideration of several interconnected parameters: thiol selectivity, conversion efficiency, bridging fidelity, payload distribution, and preservation of native antibody assembly. SDS-PAGE provides a rapid and accessible estimate of bridge reconstruction efficiency by monitoring intact IgG *versus* free chain-derived fragments, making it useful for routine screening and optimization. However, mass spectrometry remains the most informative method because it directly identifies successful bridge formation, incomplete conjugation, hydrolyzed intermediates, scrambling events, and DAR distributions with high structural resolution. Broad or unexpected mass patterns generally indicate inefficient capture of the second cysteine or competing intermolecular reactions. Accordingly, the most effective rebridging reagents are those that combine high cysteine selectivity with rapid intramolecular bridge closure, thereby minimizing the kinetic window for scrambling and incomplete conversion.

Current evidence further indicates that rebridging efficiency is strongly dependent on antibody isotype and hinge-region architecture.^15^ Importantly, this largely mirrors current clinical ADC practice, where the overwhelming majority of approved conjugates are based on human IgG1 antibodies, while only a limited number employ IgG4 scaffolds, such as Besponsa® and Mylotarg®. Human IgG1 remains the dominant ADC format because of its favorable stability, manufacturability, and well-established conjugation behavior, including efficient and reproducible interchain disulfide modification. Consistent with this, most disulfide rebridging studies have demonstrated robust and homogeneous conjugation primarily on IgG1 antibodies, whereas IgG4 and several non-human subclasses frequently show lower conversion, broader DAR distributions, or increased heterogeneity. These differences are closely linked to hinge-region structure, cysteine accessibility, and disulfide geometry, all of which strongly influence reduction and bridge reconstruction efficiency. Thus, the current focus of rebridging chemistry on IgG1 antibodies is not merely a technical limitation but closely aligns with the dominant clinical and industrial direction of ADC development, where IgG1 scaffolds continue to represent the principal therapeutic platform.^24^

## 3. The chemical toolbox of disulfide rebridging

The interchain disulfide-bridging was first disclosed in 1990 by Lawton and co-workers^26,27^ as a site-selective modification methodology. The interchain disulfide bridges are reduced and rebridged by inserting a bivalent small molecule. The advantage of this rebridging method is that native, easily produced antibodies can be used and optimal antibody conjugates with DAR4 can be synthesized with high homogeneity.^28^

**Fig. 2:**
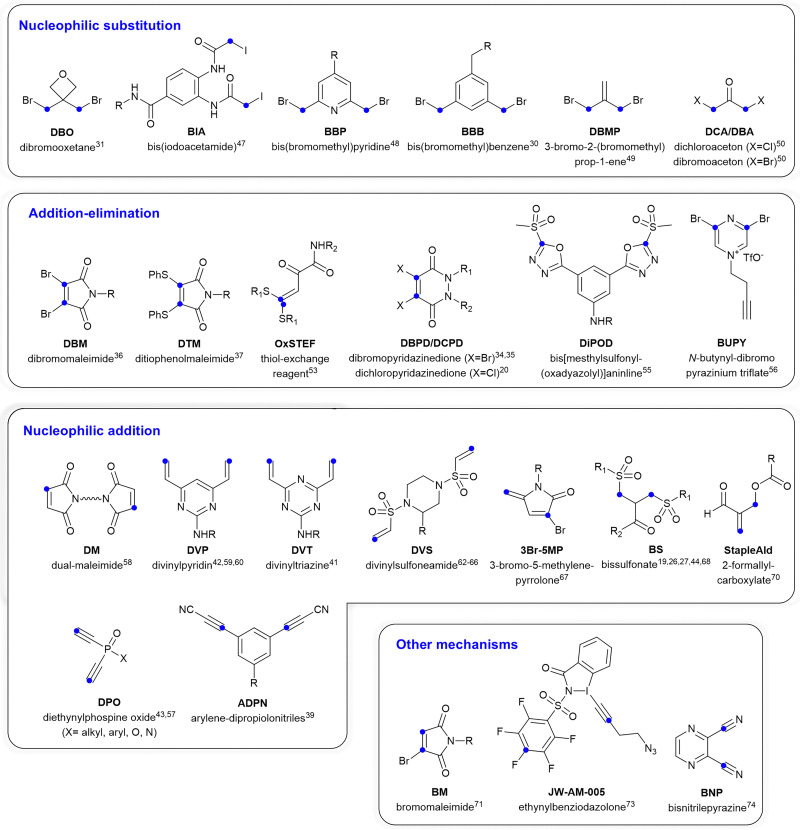
Schematic representation of rebridging agents categorized by their underlying mechanisms. The figure summarizes reported chemotypes according to their mechanisms of action, including nucleophilic substitution, addition–elimination, nucleophilic addition, and other related mechanistic pathways. Thiol attachment sites, corresponding to cysteine conjugation points, are highlighted as blue dots.

Recently, several rebridging agents were published, reacting by different mechanisms to form sulfur-carbon bonds (see Fig. 2 and Table S1). These molecular tools react by nucleophilic substitution,^29–31^ addition–elimination,^32–37^ and others by nucleophilic addition.^38–44^ These rebridging agents are typically prepared *via* a multi-step synthesis and often demand expensive starting materials.^45,46^ This underscores the need for rebridging reagents that can be synthesized *via* more feasible routes or preferably obtained directly as commercially available compounds. Beyond these dominant mechanisms, several alternative strategies have also been reported that rely on distinct reactivity patterns or multi-step transformations. These less common approaches, although mechanistically diverse, similarly aim to achieve efficient disulfide rebridging while maintaining antibody structural integrity and enabling controlled payload incorporation. In the following sections, various rebridging chemistries are discussed according to their mechanistic classes.

The application of different rebridging agents typically involves three strategies. The sequence of reduction and rebridging can control misbridging and side reactions between rebridging and reduction agents. The stepwise method with intermediate purification starts with reduction and the removal of the reducing agent by buffer exchange. Next comes the rebridging, followed by a last buffer exchange. If the rebridging agent is inert toward the reducing agent, the intermediate buffer exchange can be omitted, allowing the two steps to be performed consecutively in the same reaction vessel. In this case, the process can be described as a two-step procedure without intermediate purification. Lastly, the simultaneous addition of the reducing and rebridging agents allows cysteine reduction and conjugation parallel that defines one-pot rebridging.^35^ These approaches collectively enable the controlled reconstruction of disulfide bonds while introducing payloads to produce functional antibody conjugates.

### 3.1 Nucleophilic substitution

Nucleophilic substitution–type (S_N_) disulfide rebridging strategies represent a mechanistically distinct yet increasingly diverse class of approaches. A key strength of this group lies in the diversity of electrophilic scaffolds investigated, ranging from dibromooxetanes^31^ to bishaloacetamides,^47^ bis(bromomethyl)-pyridines,^48^ bis(bromomethyl)benzenes,^30^ allylic bromides such as 3-bromo-2-(bromomethyl)prop-1-ene,^49^ and dihaloacetones.^50^

Dibromooxetanes demonstrate excellent intrinsic reactivity, achieving >95% conversion on trastuzumab Fab, although DOL values are not explicitly reported.^31^ Bishaloacetamides, as highlighted in a recent work,^47^ enable the conjugation of large nanoparticle payloads (*e.g.*, Eudragit L-100) to atezolizumab with an average coupling efficiency of 74%. The reported regioselectivity here between hinge and interchain disulfides appears to be mostly driven by the reduction protocol employed prior to rebridging. This example highlights the critical role of antibody reduction conditions in determining the final conjugate distribution, which should be carefully optimized alongside the rebridging chemistry to achieve the desired product profile. Among benzylic systems, di(bromomethyl)-pyridines stand out for achieving an average DOL of 3.95 in brentuximab–MMAE conjugates. The resulting purified product is reasonably homogeneous (80% DAR4 by nSEC-MS).^48^ Bis(bromomethyl)-benzenes extend this chemistry to other antibody formats such as anti-CD4 and anti-CD13, though detailed DOL data are lacking.^30^ A closely related and industrially validated approach is provided by dibromomethyl heteroaromatic reagents, which have been translated into the C-Lock™ proprietary rebridging platform, licensed by Sorrento Therapeutics. In this system, activated heteroaryl dibromomethyl scaffolds (*e.g.*, dibromomethylquinoxaline derivatives) act as bis-electrophiles that rebridge reduced interchain disulfides, yielding stable conjugates with high DAR homogeneity, typically >90% DAR4 formation under mild conditions. The platform has been broadly applied to clinically relevant ADCs, including STI-6129,^51^ an anti-CD38–duostatin conjugate that has reached Phase I clinical evaluation, as well as the preclinically validated ZV0508,^52^ an anti-5T4–duostatin ADC that demonstrated efficient rebridging (>90% DAR4 by HIC), potent *in vivo* efficacy, and improved performance relative to a maleimide-linked analogue. Across these examples, C-Lock™ conjugates consistently show high conversion efficiency and strong pharmacological activity in xenograft models, while analytical studies (CE-SDS) have revealed product heterogeneity, including a minor population of half-antibody species, a feature shared with other disulfide rebridging modalities. A ready-to-use drug-linker compatible with this platform (STI-8811) is commercially available at MedChemExpress (see Table S2).

Allylic systems such as 3-bromo-2-(bromomethyl)prop-1-ene^49^ reveal a limitation of nucleophilic substitution pathways, as product distributions are strongly influenced by antibody context. Based on densitometric analysis of reported SDS-PAGE gels, the overall conversion reaches 89% for trastuzumab but only 65% for rituximab, highlighting pronounced antibody-dependent differences in rebridging efficiency. Dihaloacetones^50^ offer a more versatile platform, spanning Fab and full antibodies and accommodating diverse payloads (fatty acids, MMAF, DM1), with DOL values ranging from 1.0 to 4.0; however, overall yields can vary widely (from >95% to as low as 30%) according to the effectiveness of the payload ligation after rebridging. Overall, nucleophilic substitution–based reagents demonstrate considerable structural diversity and payload compatibility, but often at the expense of consistent full-antibody rebridging and predictable DOL control relative to the ideal value of 4.0.

### 3.2 Addition–elimination

Addition–elimination rebridging chemistries remain the most established and widely optimized class, generally proceeding through sequential nucleophilic addition and elimination steps with high selectivity and more predictable outcomes. Next generation maleimides (NGMs) are among the most prominent representatives, combining synthetic accessibility (typically 2–5 steps) with high thiol reactivity. Their well-documented susceptibility to thiol exchange can be mitigated through rapid post-conjugation hydrolysis at mildly basic pH, improving serum stability. Classical dibromomaleimides^36^ already demonstrate the capacity to approach optimal loading, with DOL values of 3.0 for fluorescent payloads and 4.1 for cytotoxic doxorubicin conjugate, indicating efficient masking of the interchain disulfide bridges. Dithiophenolmaleimides^37^ further refine this scaffold, delivering high conversions (89–99%) and DOL values in the 3.0–3.8 range, thus approaching the desired homogeneity. In contrast to many other disulfide rebridging reagents, which typically require in-house synthesis or custom chemical outsourcing, next-generation maleimide (NGM) derivatives are commercially available in ready-to-conjugate formats (see Table S2). These systems are based on a dibromomaleimide (DBM) core functionalized with bioorthogonal handles (Table S2), enabling straightforward payload diversification. A range of commonly available reactive tags includes azide, bicyclo[6.1.0]nonyne (BCN), and DABCO (1,4-diazabicyclo[2.2.2]octane), allowing modular introduction of imaging or therapeutic functions *via* subsequent bioorthogonal steps. Notably, among these commercially accessible derivatives, only a single DBM configuration is currently available as a fully preassembled drug-linker system, namely the DBM–PEG4–vcMMAE construct, which already incorporates a cytotoxic payload with enzyme cleavable peptide linker for direct ADC production. Built upon the vast accumulated advancements with NGMs, Mabwell Bioscience's IDconnect™ platform represents a next-generation cysteine conjugation strategy built on fast-hydrolyzing next-generation maleimide (NGM) chemistry. The key innovation is the use of a hydrolyzable NGM scaffold, which rapidly converts the initial succinimide thioether into a ring-opened hydrolyzed form, thereby strongly suppressing the otherwise common retro-Michael deconjugation and thiol exchange reactions that limit first-generation maleimide ADC stability. This effectively “locks in” the conjugation, yielding a more stable and homogeneous ADC profile with improved *in vivo* persistence. In Mabwell's implementation, this chemistry has been applied to clinically advanced ADC candidates, most notably Mabwell Bioscience's bulumtatug fuvedotin, which is frequently cited as one of the most advanced ADCs emerging from this platform. In preclinical and translational development reports, IDconnect™-derived ADCs consistently show high DAR homogeneity (often DAR4-enriched species depending on construct design), improved serum stability, and reduced payload loss, supporting their progression toward late-stage clinical evaluation. Among current industrial ADC platforms, this has positioned Mabwell's system as one of the most refined implementations of stabilized maleimide-based conjugation chemistry.

Similarly to dithiophenol-type NGMs, rebridging agent OxSTEF reacts *via* addition–elimination mechanism. These reagents^53^ are derived from earlier STEF reagents which operate *via* a cysteine-selective thiol-exchange mechanism followed by an irreversible sulfur-to-nitrogen acyl migration. OxSTEF reagents, however, avoid lysine modification due to their reduced reactivity. Having said that, their application to antibody–drug conjugates (ADCs) results in moderate conversions, typically 30–48% for full antibodies and 29–48% for half-antibodies. A notable feature is their susceptibility to glutathione-mediated cleavage under tumor-relevant conditions, which can be exploited for targeted payload release but may also pose off-target risks. This reactivity can be stabilized by secondary functionalization, such as oxime ligation, demonstrated in peptide conjugates but not yet translated to ADCs. Dibromopyridazinediones (DBPDs),^34,35^ designed as more stable analogues of dibromomaleimides, retain these favorable characteristics while improving robustness; they routinely achieve DOL values between 4.0 and 4.2 on trastuzumab and even enable dual-payload conjugation (*e.g.*, doxorubicin and sulfo-Cy5) without compromising stoichiometry. Their rebridging is efficient in both formats: full antibodies typically reach DOL values consistent with rebridging of all available interchain disulfides, while Fab fragments give DOL ∼1.0, reflecting the reduced number of accessible disulfides. The superiority of bromine leaving groups is clearly demonstrated by dichloropyridazinediones,^20^ which produce approximately equal amounts of full and half-antibody (49% *vs.* 51%). Building on the expanding toolbox of pyridazinedione-based chemistries, dibromopyridazinediones (DBPDs) have recently moved toward commercial availability. In addition to standard single bioorthogonal handles such as bicyclo[6.1.0]nonyne (BCN), dibenzocyclooctyne-amine (DBCO), tetrazine, and azide, DBPD platforms are uniquely available in dual-orthogonal formats, including BCN/alkyne and tetrazine/azide combinations (see Table S2). These dual-functional reagents provide a direct route toward more complex next-generation architectures, including bispecific and dual-payload ADCs,^33,54^ where multi-step conjugation strategies become feasible within a single rebridging scaffold. Other reagents such as DiPODSs^55^ extend the scope further, achieving >95% conversion on trastuzumab Fab with DOL ∼1.0 (measured by MALDI-TOF MS), consistent with selective single disulfide targeting. Taken together, these addition–elimination chemistries (particularly NGMs and DBPDs) offer the most reliable route toward homogeneous ADCs with DOL values close to the ideal 4.0, while also accommodating a wide range of payloads and antibody formats. Additionally, aromatic substitution (S_N_Ar) has also appeared as an available chemical mechanism in disulfide rebridging, exemplified by dibromopyrazinium (BUPY) reagents.^56^ These systems leverage the electron-deficient aromatic core to enable stepwise thiol substitution, offering a distinct reactivity profile compared to classical aliphatic electrophiles. In the context of trastuzumab Fab, dibromopyraziniums achieve excellent conversion (>95%) with DOL 1.0, demonstrating efficient and selective rebridging at the single disulfide level. Extension to full antibodies reveals a more nuanced picture, as payload-free systems show significant half-antibody formation (47% full *vs.* 46% half-antibody), yet the introduction of payloads such as TAMRA or DM1 resulted in efficient rebridging with DOL values of ∼4.0–4.1. The ability of dibromopyraziniums to accommodate both fluorescent and cytotoxic payloads while achieving near-ideal DOL highlights their growing potential as next-generation rebridging agents, having recently become commercially available (see Table S2).

### 3.3 Nucleophilic addition

Nucleophilic addition (Ad_N_) strategies for disulfide rebridging rely on the direct attack of cysteine thiols onto activated unsaturated systems, most prominently alkynes and electron-deficient multiple bonds, without the need for a leaving group. Among these, phosphorus-based electrophiles have been extensively studied and represent a chemically rich platform. Diethynyl-triazolyl phosphine oxides^57^ constitute one of the most advanced examples, enabling efficient rebridging of trastuzumab with cytotoxic payloads such as MMAE, reaching DOL values close to the theoretical optimum (3.8–4.0), while also supporting fluorescent conjugates, albeit with reduced selectivity as evidenced by significant half-antibody formation (57% full antibody, 35% half-antibody). Closely related diethynyl phosphinates^43^ also support efficient conjugation, with DOL values approaching 4, further confirming the suitability of activated alkynes for dual thiol capture. In contrast, alkyne phosphonamidates^43^ represent a less effective subclass, only forming half-antibody species, and doing so with quite poor conversion (∼33%). This limitation arises from the intrinsic reactivity mismatch, as typically only one thiol reacts efficiently with the unsaturated system. Arylene-dipropiolonitriles (ADPNs)^39^ overcome some of these drawbacks by providing a more rigid and electronically tuned scaffold; in particular, they achieve DOL values between 4.0 and 4.5 for trastuzumab–MMAE conjugates, placing them among the most effective Ad_N_-based systems. Overall, nucleophilic addition strategies offer a highly modular platform with broad payload compatibility, but balanced electrophilicity is needed for the sequential capture of both thiols without generating excessive half-antibody species.

Michael-type nucleophilic addition represents a specialized and highly developed subset of Ad_N_ chemistry, and arguably the most widely exploited paradigm in cysteine-selective bioconjugation. However, the main disadvantage of Michael-type nucleophilic addition agents is the reversibility of the conjugation reaction which may cause instability or require additional chemical steps to avoid the undesirable nonspecific payload-release. Maleimides remain the archetypal example, combining high reactivity with operational simplicity, although their susceptibility to retro-Michael reactions remains a known liability. Nevertheless, advanced constructs such as the dual maleimide-containing SG3710 payload^58^ demonstrate the full potential of this approach, enabling near-quantitative rebridging of trastuzumab Fab (>97% conversion) with the expected DOL of 1.0 for a single disulfide. Beyond maleimides, divinyl systems, particularly divinylpyrimidines,^42,59,60^ have emerged as highly reliable reagents, consistently affording homogeneous trastuzumab conjugates with DOL values tightly clustered around 4.0 across a wide range of payloads, including Alexa Fluor dyes, MMAE, TAMRA, and doxorubicin. Even in dual-payload settings, these reagents maintain excellent control over stoichiometry, while more sophisticated architectures incorporating multiple divinylpyrimidine^28^ units enable global antibody rebridging with sub-stoichiometric DOL values (0.76–0.90). In contrast, divinyltriazines,^41^ despite structural similarity, show inferior performance, producing predominantly half-antibody species (*e.g.*, 63%) even when the formal DOL approaches 4.0, underscoring the sensitivity of Michael acceptor reactivity to heteroaromatic substitution patterns. Notably, tetra-divinylpyrimidine (tetraDVP) scaffolds were shown to simultaneously reconnect all four interchain disulfides of an IgG1 antibody through a single linker molecule,^61^ thereby enabling the preparation of highly homogeneous DAR = 1 ADCs directly from native antibodies without the need for antibody engineering or asymmetric payload constructs. Importantly, despite the considerable structural complexity of this eight-cysteine rebridging process, conjugation efficiency and product homogeneity were largely preserved, with minimal formation of half-antibody species. Vinylsulfonamides^62–66^ further exemplify the tunability of Michael acceptors, enabling DOL values ranging from below 1 to as high as ∼7.7 depending on the antibody (trastuzumab, bevacizumab, cetuximab), payload (MMAE, fluorescein, triptolide, combretastatin A4), and reaction conditions; however, this breadth comes at the cost of product heterogeneity, often yielding mixtures of DOL 0, 2, and 4 species. Additional electrophiles such as 3-bromo-5-methylene pyrrolones^67^ expand the scope to alternative activated olefins, though typically yielding lower DOL values. A particularly important subclass is represented by bissulfonates,^19,26,27,44,68^ which were historically classified separately but undergo elimination to generate reactive intermediates *in situ* and thereby engaging in Michael-type addition. These reagents form the basis of the ThioBridge® disulfide rebridging platform,^69^ currently licensed by Abzena and has led to the development of multiple ADCs currently in clinical trials. ThioBridge® conjugation typically affords controlled DAR products with conversions in the range of 70–95%. Moreover, the resulting conjugates exhibit improved plasma stability relative to conventional maleimide linkages, while the modular reagent architecture enables incorporation of branched payloads to afford PEGylated side chains, high-DAR constructs, dual-payload and bispecific formats. Nevertheless, bissulfone-based reagents have been applied across multiple antibody formats, including murine IgG subclasses and trastuzumab, and generally provide high conversion (>88–95%) with DOL values spanning 2.3–4.0 but often not reaching the ideal value of 4.0. Although early bissulfonate reagents suffered from limited aqueous solubility and relatively slow reaction kinetics, subsequent generations have substantially improved these properties, contributing to the emergence of commercially advanced and clinically validated disulfide rebridging technologies such as ThioBridge® and related StapleAld-derived systems. StapleAld,^70^ based on unsaturated aldehyde chemistry, exhibits enhanced reactivity and reduced half-antibody formation (14–27%) while delivering DOL values in the 2.3–4.0 range and enabling further functionalization *via* oxime ligation or Wittig chemistry. Taken together, Michael-type nucleophilic addition offers a versatile and tunable framework for ADC construction, capable of delivering homogeneous conjugates and functionally diverse architectures.

### 3.4 Other disulfide rebridging mechanisms

Several rebridging strategies fall outside the classical categories of substitution or addition, representing innovative chemical technologies with distinct mechanistic features. These include photochemical approaches,^40,71^ organometallic chemistry,^72^ and multi-step^71,73^ or ring-closing strategies.^74^ Photochemical rebridging approaches provide rapid conjugation times but often suffer from limited yields. For example, bromomaleimide-mediated [2 + 2] photocyclo-addition^71^ enabled Fab rebridging in only 5 minutes of UV irradiation, achieving 42% conversion. A photoactivated Michael-type addition^40^ similarly allowed Fab rebridging of M14-G07 antibodies under prolonged UV irradiation but reached only ∼40% conversion. While these strategies are mechanistically intriguing and allow precise temporal control, their yields remain suboptimal relative to traditional rebridging agents. Ethynylbenziodazolone (JW-AM-005)^73^ represents a hybrid mechanism combining nucleophilic substitution and addition. It features dual thiol-reactive sites, a Cu(i)-cleavable linker, and an azide handle for further functionalization. This reagent has successfully rebridged multiple Fab fragments, including trastuzumab, avelumab, bevacizumab, and rituximab, with conversions of 46–84% and DOLs approaching 1.0. Its high versatility and reproducibility across several antibodies highlight it as a particularly promising agent, though synthetic accessibility remains a limitation due to its multi-step preparation and purification requirements. Organometallic Pt(ii)-based reagents^72^ exploit metal coordination for antibody rebridging and allow multiple payloads to be incorporated per disulfide bond. For trastuzumab and cetuximab Fab fragments, reactions achieved DOLs of 0.6–1.9, with conversion rates up to 95% for Fab conjugates. While this approach can maximize payload density, careful control is needed to avoid aggregation, and broader application is limited by reagent availability and complexity.

Ring-closure strategies, such as bisnitrile-mediated rebridging,^74^ offer yet another pathway, where cyclic structures stabilize the conjugate. These have been applied to trastuzumab and its Fab fragments, sometimes incorporating fluorescent payloads, but reported yields and DOLs remain underexplored. In summary, these methods demonstrate broad mechanistic diversity, from selective thiol exchange to photochemical, organometallic, cyclization, and hybrid photocyclization strategies. While some agents, like ethynylbenziodazolone, show high versatility and good conversions, others still require further optimization.

## 4. *In vitro* and *in vivo* validation of disulfide rebridged ADCs

The biological performance of rebridged ADCs is typically evaluated through a combination of *in vitro* cytotoxicity assays and *in vivo* tumor models (see Table 1). Several studies have reported that ADCs generated using disulfide rebridging strategies exhibit strong antiproliferative activity in tumor cells, frequently achieving cytotoxicity in the picomolar range when conjugated to potent payloads such as MMAE. Beyond cell-based assays, promising ADC constructs are further evaluated *in vivo* using xenograft tumor models. These studies provide insight into tumor growth inhibition, pharmacokinetics, stability, and therapeutic efficacy of the conjugates. Several rebridging chemistries, including pyridazinedione-based linkers and other disulfide rebridging strategies, have demonstrated the ability to generate ADCs inducing significant tumor regression in preclinical models.^55^ In addition to classical disulfide rebridging strategies, recent advances have introduced alternative linker chemistries that resulted in stable antibody conjugates. Modular rebridging approaches and next-generation linker systems have been shown to produce biologically active ADCs with improved stability and controlled conjugation sites.^74^ These developments further highlight the potential of rebridging strategies to support the design of homogeneous and efficient ADCs. Collectively, these studies demonstrate that rebridging chemistries provide not only precise control over antibody modification but also translate into therapeutically meaningful outcomes.

**Table 1:** Antibody–drug conjugates generated by disulfide rebridging strategies and their biological evaluation

Rebridging agent	Antibody/target	Payload	*In vitro* model	IC_50_ range	*In vivo* model	Ref.
DBM [dibromomaleimide]	Trastuzumab/HER2	Monomethyl auristatin F (MMAF)	SKOV-3; BT474	SKOV-3 : 0.01 nM; BT474 : 0.05 nM	SKOV-3 human ovarian cancer xenograft	75
DBM [dibromomaleimide]	IGNX/CD98	Monomethyl auristatin F (MMAF)	H446	0.05 nM	H446 human lung cancer xenograft	75
DTM [dithiophenol- maleimide]	Trastuzumab scFv/HER2	Monomethyl auristatin F (MMAF) with non-cleavable linker	SKBR-3	0.32 nM	—	76
DTM [dithiophenol- maleimide]	Trastuzumab scFv/HER2	Monomethyl auristatin F (MMAF) with enzyme-cleavable linker	SKBR-3	0.68 nM	—	76
DBM [dibromomaleimide]	Trastuzumab/HER2	Monomethyl auristatin E (MMAE)	BT-474	0.71 nM	—	77
DTM [dithiophenol- maleimide]	Trastuzumab/HER2	Monomethyl auristatin E (MMAE)	BT-474	1.62 nM	—	77
NGM [next generation maleimide]	Trastuzumab/HER2	Monomethyl auristatin E (MMAE)	BT-474	0.30 nM	—	77
DBPD [dibromo-pyridazinedione]	Trastuzumab/HER2	Monomethyl auristatin E (MMAE)	HCC1954	0.03-2 nM (depending on linker structure)	HCC1954 human breast cancer xenograft	78
DBPD [dibromo-pyridazinedione]	Trastuzumab Fab/HER2	Doxorubicin (DOX)	BT-474	2.8 µM	—	34
DBPD [dibromo-pyridazinedione]	Trastuzumab/HER2	Doxorubicin (DOX)	BT-474	2.1 µM	-	34
BUPY [dibromopyrazinium]	Trastuzumab/HER2	Mertansine (DM1)	SKOV-3	66 nM	—	56
DM [dual-maleimide]	Trastuzumab Fab/HER2	SG3710	SKBR-3; MDA-MB-453	SKBR-3 : 0.008 nM MDA-MB-453 : 0.172 nM	—	58
DVP [divinylpyrimidine]	Trastuzumab/HER2	Monomethyl auristatin E (MMAE)	SKBR3	0.026 nM	—	59
DVP [divinylpyrimidine]	Trastuzumab/HER2	Monomethyl auristatin E (MMAE) with non-cleavable linker	SKBR3; BT474	SKBR3 ∼30–50 nM^a^; BT474 ∼70–100 nM^a^	BT474 human breast cancer xenograft	60
DVP [divinylpyrimidine]	Trastuzumab/HER2	Monomethyl auristatin E (MMAE) with enzyme-cleavable linker	SKBR3; BT474	SKBR3 ∼20–30 nM^a^; BT474 ∼50–700 nM^a^	BT474 human breast cancer xenograft	60
DVS [N-phenyl-divinylsulfonamide]	Cetuximab/EGFR	Monomethyl auristatin E (MMAE)	HCC827	0.5 nM	HCC827 human lung cancer xenograft	66
DVS [N-phenyl-divinylsulfonamide]	Trastuzumab/HER2	Monomethyl auristatin E (MMAE)	SKBR-3	4.8 nM		64
DVS [N-phenyl-divinylsulfonamide]	Cetuximab/EGFR	Combretastatin A4 (CA4)	HCC827	4.0 nM	NCI-H1975-GFP human lung cancer xenograft	64
ADPN [arylene-dipropiolonitrile]	Trastuzumab/HER2	MMAE	SKBR-3	0.019 nM	—	39
DPO [diethynyl-phosphine oxide]	Trastuzumab/HER2	Monomethyl auristatin E (MMAE)	SKBR-3	0.1 nM	—	57
BVP [bis(vinylsulfonyl)piperazine]	mAb 401/B7-H3 (CD276)	Monomethyl auristatin E (MMAE)	U87;U251	U87 : 13.2 nM;U251 : 35.5 nM	U87 human glioblastoma cancer xenograft	79
BS [bissulfonate]	Trastuzumab Fab/HER2	Monomethyl auristatin E (MMAE)	SKBR3;BT474	SKBR3 0.2 nM;BT474 0.5 nM	—	44
BS [bissulfonate]	Trastuzumab/HER2	Monomethyl auristatin E (MMAE)	SKBR3; BT474	SKBR3 0.08 nM; BT474 0.13 nM	BT-474 human breast cancer xenograft	44
BS [bissulfonate]	Trastuzumab/HER2	Monomethyl auristatin E (MMAE)	SKBR-3	0.04 nM	BT-474 and JIMT-1 human breast cancer xenografts	19

a IC_50_ values are estimated based on a visual extraction from Fig. 2e and f of Walsh *et al.*,^60^ as exact numerical values were not reported in the original publication.

Next-generation maleimide (NGM) linkers capable of cysteine rebridging (most prominently dibromomaleimide (DBM) and dithiophenolmaleimide (DTM) derivatives) have demonstrated markedly improved biological performance of antibody–drug conjugates (ADCs) through enhanced stability and homogeneity.^75,80^ Focusing on DBM systems, its MMAF-coupled derivative has been evaluated across multiple antibody conjugates (*e.g.*, HER2- and CD98-targeting antibodies applied to breast, lung, and ovarian cancer models), thereby underscoring the generality of this rebridging approach across different cancer models. *In vitro*, DBM-based ADCs retain antigen binding and exhibit high potency and strict antigen-dependent cytotoxicity, with low nanomolar activity selectively against antigen-positive cell lines, while sparing antigen-negative controls; importantly, their activity is comparable to that of conventional maleimide ADCs.^75^*In vivo*, however, DBM rebridged ADCs show a more pronounced advantage: in xenograft models of ovarian (SKOV3) tumor, they induce superior tumor growth inhibition and complete remissions, outperforming both unconjugated antibodies and conventional maleimide-based ADC analogues. Parallel developments with DTM linkers further validate the NGM concept: DTM-based ADCs^80^ and even fragment format conjugates (including scFv constructs)^76^ achieve subnanomolar potency (∼0.3–0.7 nM) with strict HER2-dependent selectivity, independent of payload (MMAE *vs.* MMAF) or linker cleavability. These systems maintain high cytotoxicity in HER2-positive SKBR-3 cells while eliminating off-target toxicity, in contrast to free payloads. Furthermore, even comparative studies have been conducted across NGM variants (dibromo, dithiophenol, and hybrid systems) and the results confirmed consistently low nanomolar IC_50_ values and superior or equivalent efficacy relative to clinical benchmarks such as trastuzumab emtansine.^77^

Another important class of rebridging agent, pyridazinediones (PD) showed exceptional serum stability over a 7-day incubation coupled with exquisite target selectivity and cytotoxicity *in vitro*.^78^ The highly homogenous DAR 4 MMAE-equipped ADCs displayed low-nanomolar to picomolar IC_50_ values against HER2-positive HCC1954 breast cancer cells, with potency attenuated by over two orders of magnitude in HER2-negative MCF-7 controls. Translating these metrics to an *in vivo* HCC1954 xenograft model, administration of the ADCs induced rapid tumor inhibition. Furthermore, *in vitro* level comparison has been conducted of DBPD-rebridged trastuzumab Fab– *versus* mAb–doxorubicin (DOX) conjugates.^34^ The *in vitro* anti-proliferative activity of the PD rebridged Fab–DOX and full-length monoclonal antibody–doxorubicin (mAb–DOX) conjugates was evaluated against BT-474 human breast cancer cell line. Following incubation, both the rebridged Fab and mAb constructs demonstrated dose-dependent cytotoxicity with IC_50_ values in the low micromolar range. While this absolute potency is notably lower than that of contemporary ADCs armed with sub-nanomolar or picomolar tubulin inhibitors (*e.g.* MMAE, DM1), these micromolar values are entirely consistent with the intrinsic pharmacological profile of anthracyclines. Crucially, these results validate the *in vitro* functional viability of the rebridging platform and confirm that both the smaller Fab fragments and the full-length mAb architectures can successfully undergo receptor-mediated internalization and subsequent payload delivery to elicit a therapeutic response.

Validating ADCs with dibromopyrazinium (BUPY) rebridging, a biomimetic assay (bovine serum, supplemented by 1 µM GSH, 37 °C, 7 days) confirmed the physiological stability of a trastuzumab–BUPY–TAMRA conjugate. Fluorescent gel-based imaging revealed no heavy/light chain dissociation and an absence of off-target fluorophore transfer to serum proteins. This extracellular stability directly translated into highly specific *in vitro* cytotoxicity, as the BUPY-rebridged construct (trastuzumab–BUPY–DM1) demonstrated superior anti-proliferative efficacy against SKOV-3 cells relative to HER2-low MDA-MB-231 controls (66 nM and 252 nM, respectively).

Fab–drug conjugates (FDCs) rebridged by the dual-maleimide pyrrolobenzodiazepine (PBD) dimer payload, SG3710, demonstrated exceptional structural integrity and targeted therapeutic efficacy.^58^ Serum stability assessments conducted in rat serum over 15 days at 37 °C revealed no measurable loss of the cytotoxic payload, underscoring the robustness of the dual-maleimide rebridging chemistry. Moreover, *in vitro* cytotoxicity assays across a spectrum of HER2-expressing breast cancer cell lines further validated the platform's potency and specificity. The trastuzumab-based FDC exhibited potent, antigen-dependent killing of high-expressing SKBR-3 cells comparable to full-length ADCs. Noteworthily, a significant 30-fold reduction in potency was observed on lower-expressing MDA-MB-453 cells, while HER2-negative MDA-MB-468 cells were not sensitive to the treatment. Isotype control conjugates confirm that the mechanism of action is strictly mediated by specific antigen binding and subsequent internalization, positioning these rebridged FDCs as a highly stable and selective alternative to traditional ADC architectures in oncology.

Yet the most advanced rebridging agent in the field working by a nucleophilic addition mechanism of action is unmistakenly the divinylpyrimidine (DVP). Demonstrating its biological relevance, cell viability assays were conducted with trastuzumab–DVP–MMAE in HER2-positive SKBR-3 and HER2-negative MCF7 cells.^59^ MMAE has previously been reported to exhibit subnanomolar IC_50_ cytotoxicity against both cell lines. Trastuzumab–DVP–MMAE exhibited IC_50_ value of 26 pM against HER2-positive cell line SKBR3. No significant cytotoxic activity against HER2-negative MCF7 cells was observed, indicating IC_50_ values more than 30 nM. DVP-mediated disulfide rebridging of trastuzumab enables the generation of ADCs with either cleavable (C) or non-cleavable (NC) linkers, both of which retain high antigen specificity and exhibit potent, target-dependent cytotoxicity. *In vitro* evaluation across HER2-positive and HER2-negative breast cancer cell lines demonstrates that both trastuzumab–DVP–cMMAE and trastuzumab–DVP–ncMMAE induce strong, dose-dependent cytotoxicity in HER2-overexpressing models (SKBR3, BT474), while maintaining a wide therapeutic window with minimal activity in HER2-negative cells (>100 nM).^60^ The cleavable construct shows only a marginal potency advantage, suggesting that linker design exerts a limited influence on overall cellular efficacy within this platform. The IC_50_ values for all those ADCs were reported in low nanomolar range (20–70 nM on SKBR-3 and 50–100nM on BT474). These data indicate broadly comparable potency between constructs, with a consistent but modest advantage for the cleavable linker, in line with its expected intracellular release mechanism. Furthermore, it has been shown that both ADCs (with cleavable/non-cleavable linkers) can translate their strong *in vitro* selectivity into high *in vivo* efficacy. In a BT474 xenograft model, the ADCs produced dose-dependent tumor responses, with no significant activity at 1 mg kg^−1^ but complete and durable tumor regression at 10–20 mg kg^−1^, in sharp contrast to trastuzumab alone, which induced only transient growth delay. Importantly, the constructs were well tolerated, with no body weight loss, no clinical toxicity, and no histopathological damage to major organs, highlighting the favorable safety profile conferred by DVP conjugation. Notably, the tetraDVP platform enabled precise modulation of DAR value highlighted the potential utility of lower DAR species as exceptionally homogeneous and well-defined ADC architectures. In cytotoxicity assays, the corresponding ADCs displayed potent, concentration-dependent killing of HER2-positive SKBR-3 and BT474 cells, while HER2-negative cell lines remained largely unaffected.^61^*In vivo*, these constructs produced dose-dependent tumor regression in BT474 xenografts, including complete responses at higher doses.

As another well-examined subclass of nucleophilic addition based rebridging agents, disulfide rebridging using divinyl sulfone (DVS) reagents provides an alternative route to site-selective ADC construction. Trastuzumab–DVS–MMAE demonstrated potent and antigen-dependent activity comparable to clinically validated ADCs.^66^*In vitro*, the DVS-rebridged conjugate exhibits strong cytotoxicity against HER2-positive SKBR-3 cells (IC_50_ = 4.8 nM), closely matching the potency of trastuzumab emtansine (IC_50_ = 6.4 nM), while showing markedly reduced activity (>500 nM) in HER2-negative cell lines, confirming a wide therapeutic window and preserved target specificity following DVS rebridging. Extending this strategy to an EGFR-targeting context, cetuximab–DVS–MMAE conjugate displays subnanomolar potency (IC_50_ = 0.5 nM) in EGFR-positive HCC827 cells, while remained inactive in EGFR-negative models (NCI-H2228) that further underscored the efficiency of receptor-mediated internalization and payload delivery. *In vivo*, the cetuximab–DVS–MMAE conjugate induced significant tumor growth inhibition in HCC827 xenografts upon repeated intravenous dosing (20 mg kg^−1^), with no observable weight loss or systemic toxicity, indicating favorable tolerability. Importantly, linker design and payload identity jointly govern the outcome of ADC treatment. This effect is further underscored by payload diversification using combretastatin A4 (CA4),^64^ where cetuximab–DVS–CA4 conjugates achieve sustained tumor regression despite free CA4 showing no efficacy at tolerated doses. This observation suggests that ADC-mediated delivery can overcome intrinsic pharmacological limitations of the payload. Notably, cetuximab–DVS–CA4 showed robust *in vivo* efficacy with clear evidence of tumor-selective necrosis on NCI-H1975-GFP human lung cancer xenograft model that is consistently accompanied by excellent tolerability, absence of body weight loss, and lack of histopathological damage to major organs. Collectively, these findings highlight that DVS-mediated rebridging yields highly potent and selective ADCs, with conjugates validating the approach across a variety of applied targeting monoclonal antibodies, linker chemistries, and delivered payloads, thus positioning DVS chemistry as a robust platform for next-generation ADC development.

Beyond DVP- and DVS-based systems, a growing class of nucleophilic addition-driven disulfide rebridging reagents supports the versatility of site-selective ADC construction, particularly in the context of trastuzumab–MMAE conjugates and related targeted platforms. For instance, diethynyl-phosphine oxide (DPO) rebridging affords exceptionally potent constructs with highly efficient intracellular payload delivery and excellent selectivity. Trastuzumab–DPO–MMAE showed sub-nanomolar activity (IC_50_ = 0.1 nM) in HER2-positive SKBR-3 cells while maintaining complete inactivity in antigen-negative models.^43^ Similarly, arylene-dipropiolonitrile (ADPN)-based rebridging enhances this trend, with trastuzumab–ADPN–MMAE displaying ultra-high potency (IC_50_ = 0.02 nM) alongside a ∼200-fold selectivity window over HER2-negative cells, positioning these linkers for maximizing therapeutic index.^39^ Beyond HER2-targeting, bis(vinylsulfonyl)piperazine (BVP) rebridging enables the construction of stable ADCs such as mAb401-BVP-MMAE,^44^ directed against B7-H3-expressing glioblastoma models. The construct showed high serum stability with minimal payload release over 21 days that was coupled to antigen-dependent cytotoxicity (IC_50_ 13–35 nM in B7-H3-positive cells *vs.* ∼240 nM in B7-H3-knockout cells), efficient tumor localization *in vivo*, and dose-dependent tumor regression culminating in complete responses at higher doses without observable systemic toxicity. Also, the efficiency and selective payload delivery of bissulfone(BS)-mediated disulfide rebridging have been validated by the construction of homogeneous MMAE-based ADCs, particularly in the context of HER2-targeting conjugates such as trastuzumab–BS–MMAE.^19,44^ Across both Fab- and mAb-based formats, these conjugates exhibit potent and antigen-selective cytotoxicity. Fab–BS–MMAE demonstrated activity comparable to free MMAE in HER2-positive SKBR-3 cells while having ∼3 orders of magnitude reduced potency in antigen-negative models. *In vivo*, both Fab (DAR ∼1) and full antibody (DAR ∼2.8) constructs demonstrated pronounced tumor growth inhibition in BT-474 xenografts. The full-length ADC showed dose-dependent responses and complete tumor regression at higher doses and markedly outperformed both the unconjugated antibody and the free drug, while maintaining excellent tolerability. Importantly, systematic evaluation of defined DAR species (1/2/3/4) further highlights a clear DAR–efficacy relationship.^19^ Increased drug loading enhanced the potency when considered on an ADC basis, that culminated in the DAR 4 conjugate as the most active species (IC_50_ = 0.04 nM on SKBR-3), with superior activity even in HER2-low models compared to trastuzumab emtansine. This trend translates to *in vivo* experiments, where DAR 3/4 conjugates produced maximal tumor growth delay and complete tumor-free survival for DAR 4 without additional toxicity liabilities. Collectively, these studies establish bissulfone rebridging as a robust and scalable conjugation strategy with precise DAR control, excellent *in vivo* stability and tunable efficacy through controlled drug loading.

## 5. Clinical pipeline of disulfide rebridged ADCs

The clinical translation of rebridging-based ADC technologies just started, but the field has clearly progressed from proof-of-concept conjugation chemistry toward human evaluation. From a biological perspective, this transition is important because it indicates that improvements in linker stability, conjugate homogeneity, and antibody integrity may support not only *in vitro* potency and *in vivo* efficacy, but also advancement into clinical development.^81^ Current clinical examples suggest that disulfide rebridging strategies are beginning to influence the next generation of ADC development^16^ against solid tumors. Furthermore, in parallel with clinical ADC candidates, several commercially available conjugation platforms have shaped the translational landscape of this field. Technologies such as IDconnect™, C-Lock™, ThioBridge®, and earlier disulfide-bridging approaches were developed to improve manufacturability, conjugate homogeneity, and control over payload installation relative to conventional stochastic cysteine conjugation.^16,46^ However, C-Lock™ and ThioBridge® approaches are more directly aligned with the rebridging field since it relies on interchain disulfide rebridging and have more visible clinical relevance through programs such as STI-6129, MBRC-101 and OBI-999. Earlier dibromomaleimide-derived strategies also remain historically important because they helped establish the feasibility of selective disulfide bridging at the protein level, thereby paving the way for subsequent rebridging platforms.^16^

These foundational developments have now culminated in the emergence of clinically advanced rebridged ADCs, demonstrating that disulfide rebridging is not merely a conceptual bioconjugation strategy but a viable therapeutic platform. Most notably, bulumtatug fuvedotin (9MW2821) has very recently advanced into Phase III clinical evaluation,^82^ representing, to the best of our current knowledge, the first disulfide-rebridged ADC platform to reach this stage of clinical development. 9MW2821 is a next-generation Nectin-4-targeting ADC developed by Mabwell Bioscences using its proprietary IDconnect™ site-specific conjugation technology.^83^ The construct comprises a humanized anti-Nectin-4 antibody conjugated to monomethyl auristatin E (MMAE) through a cleavable valine–citrulline linker and a hydrolysable disubstituted maleimide scaffold enabling interchain cysteine rebridging, thereby generating a highly homogeneous DAR4 product. Preclinical studies demonstrated efficient Nectin-4-dependent internalization, potent bystander killing activity, and broad antitumor efficacy across multiple xenograft and patient-derived tumor models, in several cases outperforming enfortumab vedotin at equivalent exposure.^83^ Importantly, the ADC also exhibited an improved tolerability profile in non-human primates relative to enfortumab vedotin, suggesting that the site-specific interchain disulfide conjugation strategy may provide a substantially enhanced therapeutic index. Early clinical studies (NCT05773937, NCT05216965) further supported this hypothesis by demonstrating encouraging antitumor activity together with manageable toxicity across multiple Nectin-4-positive solid tumors, including urothelial, cervical, and esophageal cancers.^84^ Collectively, these findings position 9MW2821 as one of the most clinically advanced and compelling examples of a site-specific disulfide-rebridged ADC platform currently in development. Other ADCs developed by Mabwell Biosciences *via* the IDconnect™ conjugation platform have already reached clinical investigations stage. In preclinical studies, including patient-derived xenograft (PDX) models harboring diverse mutations, 7MW4911 induced strong antitumor activity, with tumor growth inhibition values ranging from 71% to 99%.^85^ Notably, the ADC demonstrated superior efficacy to both MMAE- and DXd-based ADCs in multidrug-resistant (MDR) models, underscoring its potential to overcome resistance-associated cancer phenotypes. Those promising preclinical studies resulted in the ongoing Phase I/II study (NCT07216560) in colorectal cancer and other advanced gastrointestinal (GI) malignancies. 9MW2921 is a novel TROP-2-targeting ADC engineered with a site-specific linker for the conjugation of Mtoxin, a new camptothecin-derived payload. Preclinical and early clinical findings (NCT05990452) suggest that 9MW2921 exhibits favorable tolerability together with effective antitumor activity in patients with advanced endometrial cancer (EC), HR+/HER2− breast cancer (BC), HER2− gastric cancer (GC), and non-squamous non-small cell lung cancer (nsq-NSCLC).^86^ The next ADC based on the IDconnect™ platform is 7MW3711,^87^ a B7-H3-directed ADC composed of a recombinant humanized anti-B7-H3 monoclonal antibody linked to a topoisomerase I inhibitor through a protease-cleavable linker. Preclinical and early clinical results (NCT06008379, NCT07466160) demonstrated promising antitumor activity of 7MW3711 in small cell lung cancer (SCLC) and squamous non-small cell lung cancer (Sq-NSCLC), while maintaining a manageable and well-tolerated safety profile.

One of another early-stage clinical translations is MBRC-101,^88^ an anti-EPHA5 ADC carrying an MMAE payload and developed using the ThioBridge® platform. The current clinical record (NCT06014658) describes MBRC-101 as a first-in-human open-label Phase I/Ib/II study in patients with advanced metastatic solid tumors refractory to standard therapy. Phase I has been successfully completed, yet Phase Ib is still ongoing. This candidate is particularly relevant as its preclinical package included concentration-dependent killing of EphA5-positive cells and potent antitumor activity, including complete tumor regression in multiple patient-derived xenograft models,^88^ thereby it provides a strong translational bridge between rebridging-enabled ADC design and clinical development.

Another important clinical phase rebridged DR5-targeting ADC is Oba01 (also referred to as OBI-01). The construct consists of the agonistic humanized anti-DR5 IgG1 antibody zaptuzumab conjugated to monomethyl auristatin E (MMAE) through a cleavable valine–citrulline linker using ThioBridge-mediated interchain disulfide rebridging chemistry. Preclinical pharmacokinetic and toxicology studies in rats and cynomolgus monkeys demonstrated favorable stability, minimal systemic payload release, and markedly improved tolerability compared with either free MMAE or the predecessor construct.^89^ Across multiple DR5-positive leukemia and pancreatic cancer models, Oba01 demonstrated potent and selective antitumor activity *in vitro* and *in vivo*, including durable complete regressions in CDX systems and strong efficacy in clinically relevant PDX models.^90^ Collectively, these findings position Oba01 as one of the most advanced examples of disulfide rebridged ADCs currently undergoing Phase I clinical evaluation (NCT06083870), with initial clinical results anticipated in 2026.

A further clinically investigated disulfide rebridged ADC was OBI-999, a Globo H-targeting construct composed of the humanized antibody OBI-888 linked to MMAE by ThioBridge linking strategy. OBI-999 entered Phase I studies (NCT0484366) based on compelling preclinical evidence, including potent dose-dependent tumor growth inhibition in breast, gastric, and pancreatic xenograft models as well as a lung cancer patient-derived xenograft model.^91^ In the published first-in-human Phase I study (NCT06514651), OBI-999 demonstrated acceptable tolerability and preliminary signs of disease stabilization in patients with advanced solid tumors.^81^

Taken together, these examples suggest that the clinical pipeline of rebridging-based ADC technologies is emerging. Bulumtatug fuvedotin (9MW2821) represents a particularly advanced case within this class, having progressed beyond early clinical evaluation and recently been announced to enter Phase III first-in-class studies, thereby providing the strongest evidence to date for late-stage clinical translation of a rebridged ADC approach. Overall, these results indicate that disulfide rebridged ADCs have moved beyond isolated proof-of-concept studies and are now entering the translational space. They now define a developing clinical landscape that is just beginning to take its genuine shape.

## 6. Outlook

Disulfide rebridging strategies have now moved beyond conceptual and preclinical development, reaching a critical inflection point where their translational potential is becoming tangible. As multiple rebridged ADCs are entering human clinical trials, early indications of robust stability, defined drug–antibody ratios, and preserved antigen-binding properties suggest that these approaches have the potential to play a significant role in next generation ADC therapeutics. In particular, the emergence of late-stage clinical activity, highlighted by the first-in-class bulumtatug fuvedotin (9MW2821) advancing into Phase III evaluation, represents a major milestone and provides the strongest evidence to date that rebridging-based constructs can progress toward clinical validation. Over the coming years, we anticipate that rebridging-based ADCs will transition from specialized experimental constructs to more routine components of the clinical pipeline, enabling structurally precise, modular, and reproducible antibody conjugates. This shift is likely to accelerate innovation across payload diversity, linker design, and combinatorial assembly strategies, while simultaneously streamlining manufacturability and safety considerations. This progression is further supported by the increasing accessibility of the underlying chemistry: dibromomaleimides (DBMs) have been commercially available for some time, and the more recent release of other reagents (DBPDs, C-Lock™, BUPY) as purchasable rebridging agents now provides chemical biologists with an expanded and readily accessible toolkit. Despite the significant progress in the development of disulfide rebridging technologies, comprehensive head-to-head comparisons under standardized experimental conditions remain limited, and further studies are required to elucidate the molecular determinants of product heterogeneity and their impact on the properties and performance of rebridged antibody conjugates. Nevertheless, considering the emerging clinical pipelines, the strong demand for advanced ADC platforms and the increasing commercial availability of rebridging agents, by bridging chemical precision with biological functionality, disulfide rebridging methodologies are set to establish themselves as a cornerstone of ADC development. The coming decade promises a genuine maturation of rebridged ADCs as viable, versatile, and clinically impactful therapeutics, shaping both research and clinical practice in oncology and beyond. Exciting times ahead.

## Author contributions

L. P., Í. K. K., B. J. K. and G. M. K. wrote and reviewed the manuscript.

## Conflicts of interest

There are no conflicts to declare.

## Supplementary Material

CB-OLF-D6CB00121A-s001

## Data Availability

No primary research results, software or code have been included, and no new data were generated or analysed as part of this review. Supplementary information (SI) is available (including a detailed overview of disulfide rebridging reagents, a summary of commercially available disulfide rebridging agents, and related references). See DOI: https://doi.org/10.1039/d6cb00121a. The data analysis scripts of this article are available at https://github.com/keserulab/warhead_finder.
