# Supplementary material for: Disulfide rebridging platforms for site-specific ADC conjugation
Source: RSC Chem Biol. 2026 Aug 11;7(10):1943–57. doi: 10.1039/d6cb00121a (PMC13482864; doi:10.1039/d6cb00121a)
Supplement: Supplementary file 1 [file CB-007-D6CB00121A-s001.pdf]

Supplementary Information (SI) for RSC Chemical Biology.
This journal is © The Royal Society of Chemistry 2026

Supplementary Information

Disulfide Rebridging Platforms for Site-Specific ADC Conjugation

László Petri,[a,b] Írisz K. Kovács,[a,c] Blanka J. Kiss[a,d] and György M. Keserű*[a,c]

[a] *Medicinal Chemistry Research Group, HUN-REN Research Centre for Natural Sciences, 2 Magyar tudósok krt, Budapest 1117, Hungary.*

[b] *Institute of Chemistry, ELTE Eötvös Loránd University, 1-3. Egyetem tér, 1053 Budapest, Hungary*

[c] *Department of Organic Chemistry and Technology, Budapest University of Technology and Economics, 4 Szt. Gellért tér, Budapest 1111, Hungary*

[d] *Department of Applied Biotechnology and Food Science, Budapest University of Technology and Economics, 4 Szt. Gellért tér, Budapest 1111, Hungary*

* Correspondence: keseru.gyorgy@ttk.hu

Supplementary Table S1. Overview of disulfide rebridging reagents used for antibody modification and antibody–drug conjugate (ADC) preparation. The table summarizes the chemical class of the rebridging agent, the underlying reaction mechanism used for disulfide rebridging, the antibody substrates to which the chemistry has been applied, and the reported conjugation efficiency. Reported rebridging conversion and the resulting degree of labelling (DOL) or drug-to-antibody ratio (DAR) values obtained after conjugation are included where available. Rebridging conversions are estimated from SDS–PAGE band intensities/densities reported in the original publication or, when not explicitly provided, approximated from the published gel images. These data should not be interpreted as a direct measure of payload loading efficiency or as a ranking of the intrinsic performance of the corresponding rebridging reagents. DOL/DAR values were determined by mass spectrometric analysis as reported in the cited references. The table also lists the type of payload coupled to the antibody (e.g., cytotoxic drugs, fluorophores, or other functional moieties) and the corresponding literature reference.

Rebridging mechanism	Rebridging agent	Antibody	Payload	Rebridging Conversion by SDS-PAGE[%]	DAR/DOL by mass spectrometry	Reference
Nucleophilic substitution	Dibromooxetanes	Trastuzumab Fab	-	>95%	n/a	1
	Bis-haloacetamides	Atezolizumab	Eudragit L-100 nanoparticle	average 74%	n/a	2
	Bis(bromomethyl)pyridines	Brentuximab	MMAE	65%	4.0	3
	Bis(bromomethyl)benzenes	HP2/6 (anti-CD4)	Fluorescein	n/a	n/a	4
		TEA 1/8 (anti-CD13)	Fluorescein	n/a	n/a	4
	3-Bromo-2-(bromomethyl)prop-1-ene	Trastuzumab	-	67% full antibody, 19% half antibody	n/a	5
		Rituximab	-	31% full antibody, 31% half antibody	n/a	5
	Dihaloacetones	Anti-VEGF Fab	C18 fatty acid group	>95%	1.0	6
		Anti-HER2 mAb	MMAF	n/a	3.2	6
		Unknown mAb "B"	DM1	n/a	4.0	6
		Fab of unknown antibody "C"	C18 fatty acid group	30%	1.0	6
Addition-elimination	Dibromomaleimides	Trastuzumab	Alexa Fluor 488	n/a	3.0	7
		Trastuzumab	Doxorubicin	n/a	4.1	7
	Dithiophenolmaleimides	Trastuzumab	Doxorubicin	89-99%	3.0-3.8	8
	OxSTEF reagents	Trastuzumab	Rhodamine	30-48% full antibody, 29-48% half antibody	n/a	35
	Dibromopyridazinediones	Trastuzumab	-	n/a	4.0-4.2	9
		Trastuzumab Fab	Sulfo-Cy5, PEG_4	80%	1.0	10
		Trastuzumab Fab	Doxorubicin, PEG_{20k}	n/a	1.0	10
		Trastuzumab	Doxorubicin, Sulfo-Cy5	n/a	4.0 for both payload	10
	Dichloropyridazinediones	Trastuzumab	-	49% full antibody, 51% half antibody	n/a	11
	DiPODSs	Trastuzumab Fab	Fluorescein	>95%	1.0	12
	Dibromopyraziniums	Trastuzumab Fab	-	>95%	1.0	13
		Trastuzumab Fab	DM1	n/a	1.1	13

Rebridging mechanism	Rebridging agent	Antibody	Payload	Rebridging Conversion by SDS-PAGE[%]	DAR/DOL by mass spectrometry	Reference
		Trastuzumab	-	47% full antibody, 46% half antibody	n/a	13
		Trastuzumab	TAMRA	n/a	4.1	13
		Trastuzumab	DM1	n/a	4.0	13
Nucleophilic addition	Diethynyl-triazolyl phosphine-oxides	Trastuzumab	MMAE	n/a	3.8-4.0	14
		Trastuzumab	Fluorescein	57% full antibody, 35% half antibody	n/a	14
	Diethynyl-phosphinates	Trastuzumab	Fluorescein	n/a	4.03	15
	Alkyne-phosphonamidates	Trastuzumab	-	33% half antibody	n/a	15
	Arylene-dipropiolonitriles	Trastuzumab	MMAE	n/a	4.0-4.5	16
	Divinylpyrimidines	Trastuzumab	AlexaFluor 488		4.1	17
		Trastuzumab	AZDye 488	n/a	4.0	17
		Trastuzumab	MMAE	n/a	n/a	17
		Trastuzumab	MMAE, TAMRA	n/a	n/a	17
		Trastuzumab	MMAE	n/a	3.89-3.91	18
		Trastuzumab	Doxorubicin	n/a	4.0	19
		Trastuzumab	AlexaFluor 488	n/a	3.89	19
		Trastuzumab	MMAE	>95%	4.0	19
		Trastuzumab	MMAE	>70% full antibody	0.76-0.90	20
	Divinyltriazines	Trastuzumab	AlexaFluor 488	24% full antibody, 63% half antibody	4.0	21
	Maleimides	Trastuzumab Fab	SG3710	>97%	1.0	22
	Vinylsulfonamides	Trastuzumab	Fluorescein	n/a	0.98-2.40	23
		Trastuzumab	MMAE	n/a	0.38-7.74	23
		Trastuzumab	MMAE	n/a	0.61-4.00	24
		Bevacizumab	MMAE	n/a	4.0	24
		Cetuximab	MMAE	n/a	4.0	24
		Cetuximab	Combrestatin A4	n/a	mixture of DOL0/2/4 species	25
		Trastuzumab	Triptolide	n/a	2.39-2.96	26
		Trastuzumab	MMAE	n/a	3.9	27
	3-Bromo-5-methylene pyrrolones	Anti-human IgG Fab	Fluorescein	n/a	1.1	28
	StapleAld	Trastuzumab	Fluorescein	63-78% full antibody, 14-27% half antibody	2.3-4.0	29
	Bissulfonates	225.28S IgG2ak murine mAb	-	n/a	n/a	30

Rebridging mechanism	Rebridging agent	Antibody	Payload	Rebridging Conversion by SDS-PAGE[%]	DAR/DOL by mass spectrometry	Reference
		5G6.4 IgG2a murine mAb	-	n/a	n/a	30
		B6.2 IgG1 murine mAb	-	n/a	n/a	31
		Trastuzumab Fab	MMAE	88-95%	n/a	32
		Trastuzumab	AlexaFluor 488		n/a	32
		Trastuzumab	MMAE	>95%	2.3-3.99	32
		Anti-CD4 Fab	PEG_{20k}	>95%	n/a	33
		Trastuzumab	MMAE		1.0-4.0	34
Photoactivated Michael-type addition	Alkynes	M14-G07 Fab	-	39.8%	n/a	36
Nucleophilic substitution and addition	Ethynylbenziodazolone (JW-AM-005)	Trastuzumab Fab	-	46-84%	0.26-1.06	37
		Avelumab Fab	-	61%	1.0	37
		Bevacizumab Fab	-	72%	0.94	37
		Rituximab Fab	-	76%	0.95	37
		Trastuzumab	-	82%	2.6	37
		Trastuzumab Fab	Biotin	65-72%	0.98-1.0	37
Organometallic chemistry	Pt(II)-based reagent	Trastuzumab Fab	Camptothecin	95%	0.6-1.9	38
		Cetuximab Fab	Camptothecin	n/a	n/a	38
		Trastuzumab	Camptothecin	n/a	2.5-8	38
		Cetuximab	Camptothecin	n/a	2.5-8	38
		Rituximab	Camptothecin	n/a	2.5-8	38
Ring closure	Bisnitriles	Trastuzumab Fab	-	n/a	n/a	39
		Trastuzumab Fab	Fluorescein	n/a	n/a	39
		Trastuzumab	Fluorescein	n/a	n/a	39
Nucleophilic substitution, followed by photocyclization	Bromomaleimides	Polyclonal anti-mouse IgG Fab	Biotin	48%	n/a	40
		CP203 Fab	Biotin	42%	n/a	40

Supplementary Table S2. Overview of commercially available disulfide rebridging agents readily available for antibody modification and antibody–drug conjugate (ADC) preparation. The table summarizes each reagent's chemical class and provides details of commercially available derivatives, including compound names and CAS numbers (where available), the incorporated bioorthogonal handles, and information on suppliers, catalog numbers, and associated hyperlinks.

Rebridging agent	Structure	Name	Handle	Supplier	Catalog No.	Hyperlink
DBM (dibromo-maleimides)	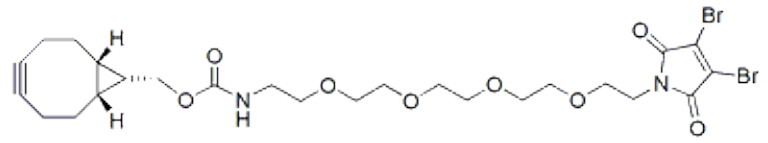	3,4-dibromomaleimide-PEG4-endo-BCN	bicyclo[6.1.0]nonyne (BCN)	BroadPharm Ltd.	BP-44526	https://broadpharm.com/product/bp-44526
	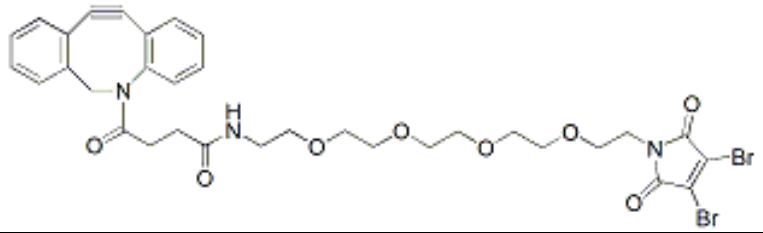	3,4-dibromomaleimide-PEG4-DBCO	Dibenzocyclooctyne-amine (DBCO)	BroadPharm Ltd.	BP-29795	https://broadpharm.com/product/bp-29795
	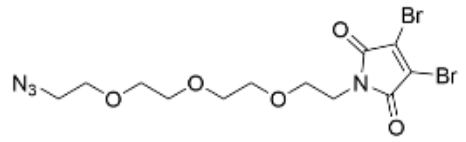	3,4-dibromomaleimide-PEG3-azide	azide	PrecisePEG Ltd.	AAD-9385	https://precisepeg.com/products/dbm-peg3-azide
	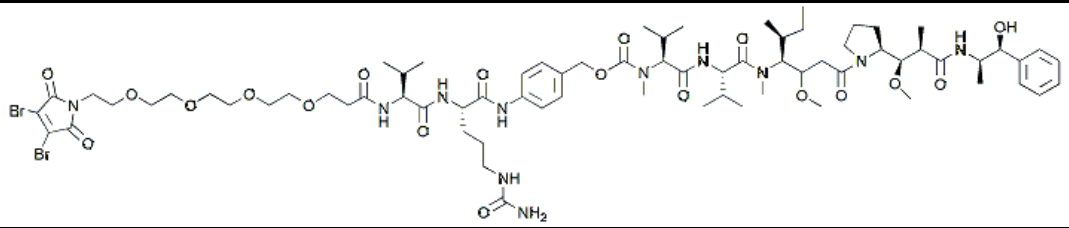	3,4-dibromomaleimide-PEG4-vcMMAE	Val-Cit-PAB-MMAE	BroadPharm Ltd.	BP-41473	https://broadpharm.com/product/bp-41473
DBPD (dibromo-pyridazinedione)	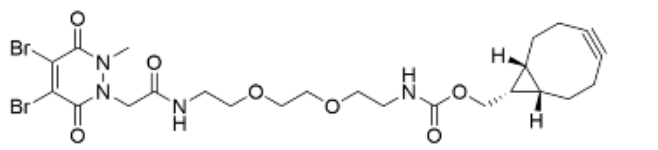	4,5-dibromo-pyridazinedione - PEG2-endo-BCN	bicyclo[6.1.0]nonyne (BCN)	PrecisePEG Ltd.	AAD-5636	https://precisepeg.com/products/br2pd-peg2-bcn
	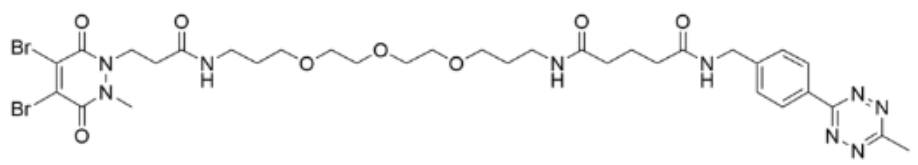	4,5-dibromo-pyridazinedione - PEG3-Me-Tet	3-methyl-1,2,4,5-tetrazine (Me-Tet)	PrecisePEG Ltd.	AAD-5637	https://precisepeg.com/products/metz-bis-nh2-c1-peg3-br2pd
	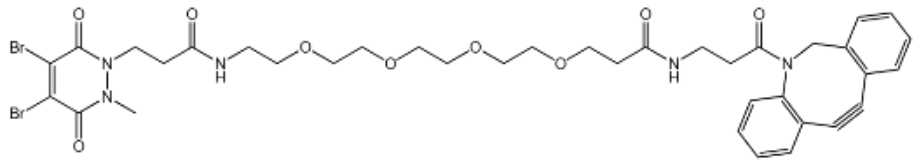	4,5-dibromo-pyridazinedione - PEG4-DBCO	dibenzocyclooctyne - amine (DBCO)	Iris Biotech Gmbh.	RL-8810	https://iris-biotech.de/global/rl-8810
	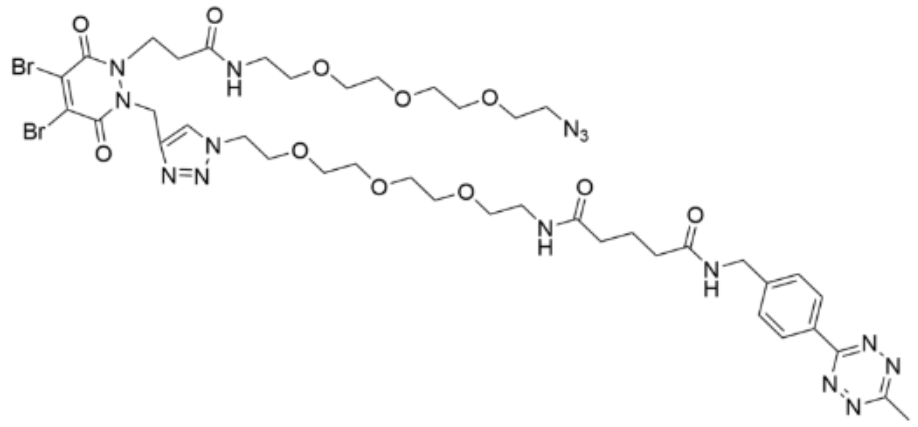	4,5-dibromo-pyridazinedione - PEG3-Me-Tet	3-methyl-1,2,4,5-tetrazine (Me-Tet) *AND* azide	PrecisePEG Ltd.	AAD-5642	https://precisepeg.com/products/mtz-peg3-triazole-br2pd-peg3-azide

Rebridging agent	Structure	Name	Handle	Supplier	Catalog No.	Hyperlink
	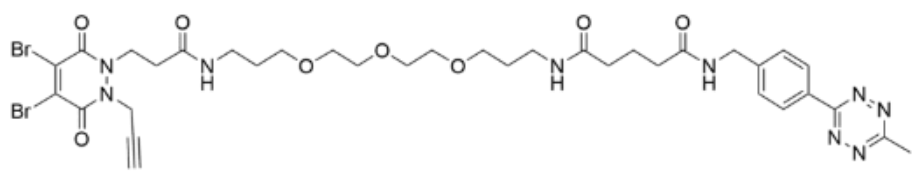	4,5-dibromo-pyridazinedione -PEG3-Me-Tet-C1-alkyne	3-methyl-1,2,4,5-tetrazine (Me-Tet) *AND* alkyne	PrecisePEG Ltd.	AAD-5638	https://precisepeg.com/products/metz-bis-nh2-c1-peg3-propargyl-br2pd
	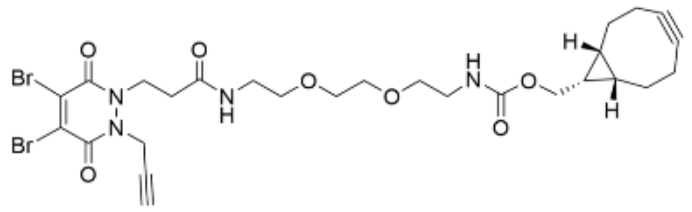	4,5-dibromo-pyridazinedione -PEG2-endo-BCN-C1-alkyne	bicyclo[6.1.0]nonyne (BCN) *AND* alkyne	PrecisePEG Ltd.	AAD-5640	https://precisepeg.com/products/bcn-peg2-propargyl-br2pd
C-Lock™	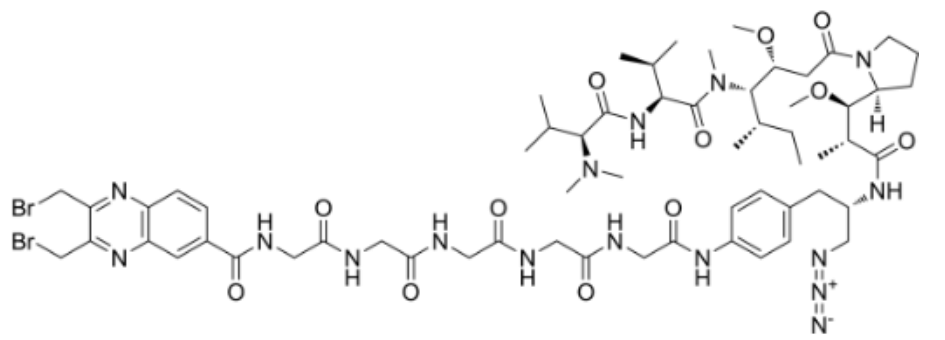	C-Lock-Gly5-Duo5	Duostatin 5	MedChemExpress LLC	HY-168550	https://www.medchemexpress.com/sti-8811-drug-linker.html
BUPY	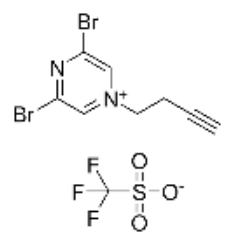	BUPY-C2-alkyne	alkyne	Merck Gmbh.	SML4282	https://www.sigmaaldrich.com/HU/hu/product/sigma/sml4282

References

1. Martínez-Sáez, N., Sun, S., Oldrini, D., Sormanni, P., Boutureira, O., Carboni, F., Compañón, I., Deery, M.J., Vendruscolo, M., Corzana, F., et al. (2017). Oxetane Grafts Installed Site-Selectively on Native Disulfides to Enhance Protein Stability and Activity In Vivo. Angew. Chem. Int. Ed. *56*, 14963–14967. https://doi.org/10.1002/anie.201708847.
2. Alkhawaja, B., Abuarqoub, D., Al-natour, M., Alshaer, W., Abdallah, Q., Esawi, E., Jaber, M., Alkhawaja, N., Ghanim, B.Y., Qinna, N., et al. (2024). Facile Rebridging Conjugation Approach to Attain Monoclonal Antibody-Targeted Nanoparticles with Enhanced Antigen Binding and Payload Delivery. Bioconjug. Chem. https://doi.org/10.1021/acs.bioconjchem.4c00275.
3. Juen, L., Baltus, C.B., Gély, C., Feuillâtre, O., Desgranges, A., Viaud-Massuard, M.-C., and Martin, C. (2021). Innovative Bioconjugation Technology for Antibody–Drug Conjugates: Proof of Concept in a CD30-Positive Lymphoma Mouse Model. Bioconjug. Chem. *32*, 595–606. https://doi.org/10.1021/acs.bioconjchem.1c00058.
4. Ramos-Tomillero, I., Perez-Chacon, G., Somovilla-Crespo, B., Sanchez-Madrid, F., Domínguez, J.M., Cuevas, C., Zapata, J.M., Rodríguez, H., and Albericio, F. (2018). Bioconjugation through Mesitylene Thiol Alkylation. Bioconjug. Chem. *29*, 1199–1208. https://doi.org/10.1021/acs.bioconjchem.7b00828.
5. Sun, S., Akkapeddi, P., Marques, M.C., Martínez-Sáez, N., Torres, V.M., Cordeiro, C., Boutureira, O., and Bernardes, G.J.L. (2019). One-pot stapling of interchain disulfides of antibodies using an isobutylene motif. Org. Biomol. Chem. *17*, 2005–2012. https://doi.org/10.1039/C8OB02877J.
6. Hu, Q., and Imase, H. (2014). Methods for making conjugates from disulfide-containing proteins.
7. Morais, M., Nunes, J.P.M., Karu, K., Forte, N., Benni, I., Smith, M.E.B., Caddick, S., Chudasama, V., and Baker, J.R. (2017). Optimisation of the dibromomaleimide (DBM) platform for native antibody conjugation by accelerated post-conjugation hydrolysis. Org. Biomol. Chem. *15*, 2947–2952. https://doi.org/10.1039/C7OB00220C.
8. Schumacher, F.F., Nunes, J.P.M., Maruani, A., Chudasama, V., Smith, M.E.B., Chester, K.A., Baker, J.R., and Caddick, S. (2014). Next generation maleimides enable the controlled assembly of antibody–drug conjugates *via* native disulfide bond bridging. Org. Biomol. Chem. *12*, 7261–7269. https://doi.org/10.1039/C4OB01550A.
9. Bahou, C., Richards, D.A., Maruani, A., Love, E.A., Javaid, F., Caddick, S., Baker, J.R., and Chudasama, V. (2018). Highly homogeneous antibody modification through optimisation of the synthesis and conjugation of functionalised dibromopyridazinediones. Org. Biomol. Chem. *16*, 1359–1366. https://doi.org/10.1039/C7OB03138F.
10. Maruani, A., Smith, M.E.B., Miranda, E., Chester, K.A., Chudasama, V., and Caddick, S. (2015). A plug-and-play approach to antibody-based therapeutics via a chemoselective dual click strategy. Nat. Commun. *6*, 6645. https://doi.org/10.1038/ncomms7645.
11. Bahou, C., Love, E.A., Leonard, S., Spears, R.J., Maruani, A., Armour, K., Baker, J.R., and Chudasama, V. (2019). Disulfide Modified IgG1: An Investigation of Biophysical Profile and Clinically Relevant Fc Interactions. Bioconjug. Chem. *30*, 1048–1054. https://doi.org/10.1021/acs.bioconjchem.9b00174.
12. Khozeimeh Sarbisheh, E., Dewaele-Le Roi, G., Shannon, W.E., Tan, S., Xu, Y., Zeglis, B.M., and Price, E.W. (2020). DiPODS: A Reagent for Site-Specific Bioconjugation via the Irreversible Rebridging of Disulfide Linkages. Bioconjug. Chem. *31*, 2789–2806. https://doi.org/10.1021/acs.bioconjchem.0c00590.
13. Szepesi Kovács, D., Pásztor, B., Ábrányi-Balogh, P., Petri, L., Imre, T., Simon, J., Tátrai, E., Várady, G., Tóvári, J., Szijj, P.A., et al. (2024). Site-Selective Antibody Conjugation with Dibromopyrazines. Bioconjug. Chem. *35*, 1373–1379. https://doi.org/10.1021/acs.bioconjchem.4c00296.
14. Stieger, C.E., McMillan, A.J., de Geus, M.A.R., Arafiles, J.V. V., Franz, L., and Hackenberger, C.P.R. (2025). Unsaturated Phosphine Oxides for Modular Antibody Rebridging and Single Reagent Peptide-Cyclization-Bioconjugation. Angew. Chem. Int. Ed. *64*. https://doi.org/10.1002/anie.202508656.
15. Stieger, C.E., Franz, L., Körlin, F., and Hackenberger, C.P.R. (2021). Diethynyl Phosphinates for Cysteine-Selective Protein Labeling and Disulfide Rebridging. Angew. Chem. Int. Ed. *60*, 15359–15364. https://doi.org/10.1002/anie.202100683.
16. Koniev, O., Dovgan, I., Renoux, B., Ehkirch, A., Eberova, J., Cianférani, S., Kolodych, S., Papot, S., and Wagner, A. (2018). Reduction–rebridging strategy for the preparation of ADPN-based antibody–drug conjugates. Medchemcomm *9*, 827–830. https://doi.org/10.1039/C8MD00141C.
17. Hanby, A.R., Walsh, S.J., Counsell, A.J., Ashman, N., Mortensen, K.T., Carroll, J.S., and Spring, D.R. (2022). Antibody dual-functionalisation enabled through a modular divinylpyrimidine disulfide rebridging strategy. Chemical Communications *58*, 9401–9404. https://doi.org/10.1039/D2CC02515A.
18. Walsh, S.J., Omarjee, S., Dannheim, F.M., Couturier, D.-L., Bexheti, D., Mendil, L., Cronshaw, G., Fewster, T., Gregg, C., Brodie, C., et al. (2022). Divinylpyrimidine reagents generate antibody–drug conjugates with excellent *in vivo* efficacy and tolerability. Chemical Communications *58*, 1962–1965. https://doi.org/10.1039/D1CC06766D.
19. Walsh, S.J., Omarjee, S., Galloway, W.R.J.D., Kwan, T.T.-L., Sore, H.F., Parker, J.S., Hyvönen, M., Carroll, J.S., and Spring, D.R. (2019). A general approach for the site-selective modification of native proteins, enabling the generation of stable and functional antibody–drug conjugates. Chem. Sci. *10*, 694–700. https://doi.org/10.1039/C8SC04645J.

20. King, T.A., Walsh, S.J., Kapun, M., Wharton, T., Krajcovicova, S., Glossop, M.S., and Spring, D.R. (2023). Disulfide re-bridging reagents for single-payload antibody-drug conjugates. Chemical Communications *59*, 9868–9871. https://doi.org/10.1039/D3CC02980H.
21. Counsell, A.J., Walsh, S.J., Robertson, N.S., Sore, H.F., and Spring, D.R. (2020). Efficient and selective antibody modification with functionalised divinyltriazines. Org. Biomol. Chem. *18*, 4739–4743. https://doi.org/10.1039/D0OB01002B.
22. Ruddle, B.T., Fleming, R., Wu, H., Gao, C., and Dimasi, N. (2019). Characterization of Disulfide Bond Rebridged Fab–Drug Conjugates Prepared Using a Dual Maleimide Pyrrolobenzodiazepine Cytotoxic Payload. ChemMedChem *14*, 1185–1195. https://doi.org/10.1002/cmdc.201900077.
23. Huang, R., Li, Z., Sheng, Y., Yu, J., Wu, Y., Zhan, Y., Chen, H., and Jiang, B. (2018). *N* -Methyl- *N* -phenylvinylsulfonamides for Cysteine-Selective Conjugation. Org. Lett. *20*, 6526–6529. https://doi.org/10.1021/acs.orglett.8b02849.
24. Wei, D., Jiang, Y., Mao, Y., Xu, Z., Chen, J., Gao, X., Li, J., Jiang, B., and Chen, H. (2023). Phenyldivinylsulfonamides for the construction of antibody–drug conjugates with controlled four payloads. Bioorg. Chem. *134*, 106463. https://doi.org/10.1016/j.bioorg.2023.106463.
25. Huang, R., Sheng, Y., Xu, Z., Wei, D., Song, X., Jiang, B., and Chen, H. (2021). Combretastatin A4-derived payloads for antibody-drug conjugates. Eur. J. Med. Chem. *216*, 113355. https://doi.org/10.1016/j.ejmech.2021.113355.
26. Wei, D., Mao, Y., Xu, Z., Chen, J., Li, J., Jiang, B., and Chen, H. (2021). Site-specific construction of triptolide-based antibody-drug conjugates. Bioorg. Med. Chem. *51*, 116497. https://doi.org/10.1016/j.bmc.2021.116497.
27. Huang, R., Sheng, Y., Wei, D., Lu, W., Xu, Z., Chen, H., and Jiang, B. (2020). Divinylsulfonamides enable the construction of homogeneous antibody–drug conjugates. Bioorg. Med. Chem. *28*, 115793. https://doi.org/10.1016/j.bmc.2020.115793.
28. Zhang, Y., Zang, C., An, G., Shang, M., Cui, Z., Chen, G., Xi, Z., and Zhou, C. (2020). Cysteine-specific protein multi-functionalization and disulfide bridging using 3-bromo-5-methylene pyrrolones. Nat. Commun. *11*, 1015. https://doi.org/10.1038/s41467-020-14757-4.
29. Shaikh, D.S., Rajput, S., and Kalia, D. (2026). Rapid and High-Yielding Disulfide Bioconjugation at Any Desired Site in Proteins. J. Am. Chem. Soc. *148*, 4032–4042. https://doi.org/10.1021/jacs.5c13581.
30. Del Rosario, R.B., Wahl, R.L., Brocchini, S.J., Lawton, R.G., and Smith, R.H. (1990). Sulfhydryl site-specific crosslinking and labeling of monoclonal antibodies by a fluorescent equilibrium transfer alkylation crosslink reagent. Bioconjug. Chem. *1*, 51–59. https://doi.org/10.1021/bc00001a006.
31. Liberatore, F.A., Comeau, R.D., McKearin, J.M., Pearson, D.A., Belonga, B.Q., Brocchini, S.J., Kath, J., Phillips, T., Oswell, K., and Lawton, R.G. (1990). Site-directed chemical modification and crosslinking of a monoclonal antibody using equilibrium transfer alkylating crosslink reagents. Bioconjug. Chem. *1*, 36–50. https://doi.org/10.1021/bc00001a005.
32. Badescu, G., Bryant, P., Bird, M., Henseleit, K., Swierkosz, J., Parekh, V., Tommasi, R., Pawlisz, E., Jurlewicz, K., Farys, M., et al. (2014). Bridging Disulfides for Stable and Defined Antibody Drug Conjugates. Bioconjug. Chem. *25*, 1124–1136. https://doi.org/10.1021/bc500148x.
33. Brocchini, S., Balan, S., Godwin, A., Choi, J.-W., Zloh, M., and Shaunak, S. (2006). PEGylation of native disulfide bonds in proteins. Nat. Protoc. *1*, 2241–2252. https://doi.org/10.1038/nprot.2006.346.
34. Bryant, P., Pabst, M., Badescu, G., Bird, M., McDowell, W., Jamieson, E., Swierkosz, J., Jurlewicz, K., Tommasi, R., Henseleit, K., et al. (2015). In Vitro and In Vivo Evaluation of Cysteine Rebridged Trastuzumab–MMAE Antibody Drug Conjugates with Defined Drug-to-Antibody Ratios. Mol. Pharm. *12*, 1872–1879. https://doi.org/10.1021/acs.molpharmaceut.5b00116.
35. Nisavic, M., Wørmer, G.J., Nielsen, C.S., Jeppesen, S.M., Palmfeldt, J., and Poulsen, T.B. (2023). oxSTEF Reagents Are Tunable and Versatile Electrophiles for Selective Disulfide-Rebridging of Native Proteins. Bioconjug. Chem. *34*, 994–1003. https://doi.org/10.1021/acs.bioconjchem.3c00005.
36. Griebenow, N., Dilmaç, A.M., Greven, S., and Bräse, S. (2016). Site-Specific Conjugation of Peptides and Proteins via Rebridging of Disulfide Bonds Using the Thiol–Yne Coupling Reaction. Bioconjug. Chem. *27*, 911–917. https://doi.org/10.1021/acs.bioconjchem.5b00682.
37. Koutsopetras, I., Mishra, A.K., Benazza, R., Hernandez-Alba, O., Cianférani, S., Chaubet, G., Nicolai, S., and Waser, J. (2023). Cysteine-Cysteine Cross-Conjugation of both Peptides and Proteins with a Bifunctional Hypervalent Iodine-Electrophilic Reagent. Chemistry – A European Journal *29*. https://doi.org/10.1002/chem.202302689.
38. Gupta, N., Kancharla, J., Kaushik, S., Ansari, A., Hossain, S., Goyal, R., Pandey, M., Sivaccumar, J., Hussain, S., Sarkar, A., et al. (2017). Development of a facile antibody–drug conjugate platform for increased stability and homogeneity. Chem. Sci. *8*, 2387–2395. https://doi.org/10.1039/C6SC05149A.
39. Patel, M., Forte, N., Bishop, C.R., Porter, M.J., Dagwell, M., Karu, K., Chudasama, V., and Baker, J.R. (2024). The Nitrile Bis-Thiol Bioconjugation Reaction. J. Am. Chem. Soc. *146*, 274–280. https://doi.org/10.1021/jacs.3c08762.
40. Matsushita, T., Maruyama, N., Koyama, T., Hatano, K., and Matsuoka, K. (2025). Functional evaluation of biotinylated Fab fragments after photochemical re-bridging with monobromomaleimide. Bioorg. Med. Chem. *129*, 118305. https://doi.org/10.1016/j.bmc.2025.118305.
